# Apolipoprotein A-I levels in the survival of patients with colorectal cancer: a retrospective study

**DOI:** 10.3389/fendo.2024.1318416

**Published:** 2024-06-11

**Authors:** Hailun Xie, Lishuang Wei, Qiwen Wang, Shuangyi Tang, Jialiang Gan

**Affiliations:** ^1^ Department of Gastrointestinal Gland Surgery, the First Affiliated Hospital, Guangxi Medical University, Nanning, Guangxi, China; ^2^ Guangxi Key Laboratory of Enhanced Recovery After Surgery for Gastrointestinal Cancer, Nanning, Guangxi, China; ^3^ Department of Geriatric Respiratory Disease Ward, the First Affiliated Hospital, Guangxi Medical University, Nanning, Guangxi, China; ^4^ Department of Colorectal and Anal Surgery, the First Affiliated Hospital, Guangxi Medical University, Nanning, Guangxi, China; ^5^ Department of Pharmacy, the First Affiliated Hospital, Guangxi Medical University, Nanning, Guangxi, China

**Keywords:** colorectal cancer, prognostic, recurrence, ApoA-I, survival

## Abstract

**Background:**

Abnormal lipid levels have been associated with cancer incidence and progression. However, limited studies have investigated the relationship between apolipoprotein A-I (ApoA-I) and colorectal cancer (CRC). This study assessed the significance of ApoA-I levels in progression-free survival (PFS) and overall survival (OS) of patients with CRC.

**Methods:**

Survival curves were compared using Kaplan–Meier analysis, while the predictive values of various lipid indicators in CRC prognosis were evaluated based on receiver operating characteristic curves. The factors influencing PFS and OS in patients with CRC were analyzed using Cox proportional hazards regression models. Finally, the relationship between ApoA-I level and disease recurrence was investigated through logistic regression analysis. The optimal Apo-I level was determined through maximally selected rank statistics.

**Results:**

Using the optimal ApoA-I cutoff value (0.9 g/L), the 1,270 patients with CRC were categorized into low (< 0.9 g/L, 275 cases) and high (≥0.9 g/L, 995 cases) ApoA-I groups. Compared with other lipid indicators, ApoA-I demonstrated superior predictive accuracy. The high ApoA-I group exhibited significantly higher survival rates than the low ApoA-I group (PFS, 64.8% vs. 45.2%, *P* < 0.001; OS, 66.1% vs. 48.6%, *P* < 0.001). Each one-standard-deviation increase in ApoA-I level was related to a 12.0% decrease in PFS risk (hazard ratio [HR] 0.880; 95% confidence interval [CI], 0.801–0.968; *P* = 0.009) and an 11.2% decrease in OS risk (HR 0.888; 95%CI, 0.806–0.978; *P* = 0.015). Logistic regression analysis revealed that patients with low ApoA-I had a 32.5% increased risk of disease recurrence (odds ratio [OR] 0.675; 95%CI, 0.481–0.946; *P* = 0.0225) compared with those with high ApoA-I. PFS/OS nomograms based on ApoA-I demonstrated excellent prognostic prediction accuracy.

**Conclusions:**

Serum ApoA-I level may be a valuable and non-invasive tool for predicting PFS and OS in patients with CRC.

## Background

Colorectal cancer (CRC) is among the most prevalent malignancies and poses a significant threat to human health. In Western countries, CRC ranks as the second leading cause of cancer-related fatalities (1). The situation in China is no less daunting, where CRC is the third leading cause of cancer-related death (2). Generally, the risk of CRC increases rapidly with age. In recent years, patients with CRC aged <65 years in the United States have accounted for 45% of cases. Patients with early-stage CRC who undergo surgical treatment can achieve a survival rate of >90%. However, 60% of CRC cases are diagnosed at an advanced stage, with a higher proportion of patients experiencing distant metastasis, leading to a survival rate of <20% for metastatic CRC (3). Therefore, widespread concern exists regarding the need to identify factors that can guide clinical treatment and predict the outcome of patients with CRC.

Numerous prognostic factors utilizing peripheral biochemical biomarkers have been validated (4–6). Dysregulated lipid levels have been consistently linked to the occurrence, risk, and progression of CRC. Dysregulation of lipid metabolism has a significant impact on the tumor microenvironment, suggesting a potential interaction between lipid metabolism and immune response (7). Increasing evidence suggests that lipid metabolism is a key determinant affecting immune therapy and clinical responses in cancer patients (8). Dysregulated lipid metabolism can enhance the occurrence, establishment, and metastatic potential of tumor cells (9). Controlling systemic lipid metabolism may contribute to improving responses to immunotherapy, with lipid metabolism interventions serving as regulators of anticancer immune responses and catalysts for anticancer immunotherapy, offering significant therapeutic potential (10). Therefore, lipid metabolism, especially that of apolipoprotein A-I (ApoA-I), and its relationship with cancer have recently attracted significant attention. ApoA-I is primarily synthesized in the liver and is a significant constituent of high-density lipoproteins (HDL). ApoA-I possesses multiple biological functions, including cholesterol transport, antioxidant properties, anti-inflammatory effects, and anticoagulant activities (11). Pulcrano et al. indicated that ApoA-I is a beneficial lipoprotein, particularly as a protective factor against cardiovascular diseases (12). ApoA-I is closely associated with the onset, progression, and prognosis of various cancer types, including renal cell carcinoma (13), esophageal squamous cell carcinoma (14), nasopharyngeal carcinoma (15), ovarian cancer (16), non-small cell lung cancer (17), and bladder cancer (18). Nevertheless, research investigating the relationship between ApoA-I and the prognosis of patients with CRC is scarce (19). The role of ApoA-I in cancer remains controversial, with some studies suggesting anti-cancer effects (20, 21) and other studies reporting a positive correlation between ApoA-I level and breast cancer risk (22).

In this context, the present study utilized clinical cohort analysis to explore the relationship between ApoA-I levels and the progression-free survival (PFS) and overall survival (OS) of patients with CRC who underwent surgical treatment. Additionally, a predictive nomogram based on ApoA-I was developed to offer novel insights into the prognostic evaluation of patients with CRC.

## Methods

### Participants

This study included 1,270 patients with CRC who received treatment at the First Affiliated Hospital of Guangxi Medical University between 2015 and 2017. The eligibility criteria for inclusion were: 1. no preoperative treatments including radiotherapy, chemotherapy, or immunotherapy; 2. postoperative pathological confirmation of CRC; 3. availability of complete clinical and pathological data; and 4. age ≥18 years. The exclusion criteria included: 1. history of dyslipidemia and 2. the presence of acute and chronic diseases such as hereditary hyperlipidemia, other malignant tumors, liver or kidney dysfunction, and cardiovascular diseases, which could potentially interfere with the study results.

### Data collection

Various clinical data, including age, sex, body mass index (BMI), medical history, serum lipid parameters, postoperative pathological examination reports (tumor location, size, TNM stage, neural/vascular invasion, and differentiation degree), postoperative radiotherapy, and chemotherapy, were collected. The assessed lipid indicators included ApoA-I, ApoB, ApoA-I/ApoB, total cholesterol (TC), triglyceride (TG), high-density lipoprotein (HDL), low-density lipoprotein (LDL), and lipoprotein (a) [Lp(a)]. The clinical and pathological data collected were obtained from the electronic medical records system of the research center.

### Follow-up

All patients were followed up through telephone consultations and regular outpatient visits until January 2023, resulting in a total follow-up period ranging from 1 month to 106 months (median 64.9 months). OS and PFS were assessed in the prognostic follow-up cohort. OS was defined as the duration from the date of surgery until either death attributed to CRC or the last follow-up, whereas PFS was defined as the time elapsed from the date of surgery to the occurrence of local or distant disease recurrence.

### Statistical analysis

Continuous data were reported as means ± standard deviation (SD) or medians (interquartile range [IQR]). T-tests were performed for comparisons between groups. Categorical data were presented as counts (%) and compared using the χ² test. The optimal threshold of ApoA-I was determined using maximally selected rank statistics. Kaplan–Meier (K–M) analysis with log-rank test was employed to compare survival curves between the low and high ApoA-I groups according to the threshold value. The area under the receiver operating characteristic (ROC) curve (AUC) was used to analyze the predictive value of various lipid indicators for CRC prognosis. To investigate the continuous association between ApoA-I and survival, restricted cubic splines (RCS) were employed. The Cox proportional hazards regression models were applied to investigate the variables that influenced both PFS and OS in patients with CRC. Logistic regression analysis was performed to evaluate the correlation between ApoA-I levels and CRC recurrence. Finally, nomograms were constructed using statistically significant indicators, and discrimination and calibration were evaluated using the C-index, ROC curve, and calibration curve. The clinical utility of the nomogram was evaluated using decision curve analysis (DCA). *P* < 0.05 was considered statistically significant.

## Results

### Comparisons of general clinical data between patients with CRC according to ApoA-I level

Among the 1,270 patients with CRC, the mean age was 59.22 ± 12.65 years, and 63.3% were male. A total of 623 patients (49.1%) had colon cancer, and 647 patients (50.9%) had rectal cancer. Recurrence occurred in 349 patients (27.5%) and 517 patients (40.7%) died. Clinicopathological staging revealed 318 cases (25.0%) of stage I–II and 952 cases (75.0%) of stage III–IV CRC.

The 1,270 patients with CRC were divided into two groups based on the optimal ApoA-I cutoff: low (ApoA-I <0.9 g/L, 275 cases) and high (ApoA-I ≥0.9 g/L, 995 cases) (**Figure S1**). ApoA-I level was significantly associated with several factors, including sex, hypertension, diabetes, T stage, M stage, tumor location, tumor size, carcinoembryonic antigen (CEA) level, length of hospital stay, and hospitalization costs. Additionally, patients with a low ApoA-I level had a significantly higher overall mortality rate than those with a high ApoA-I level, with a difference of 16.7% (53.8% vs. 37.1%; *P* < 0.001) (**Table S1**). Furthermore, the investigation of the distribution of median ApoA-I levels among various clinicopathological characteristics revealed notably lower ApoA-I levels in male patients with stage III–IV CRC, patients who experienced recurrence, and patients who died (**Figure S2**).

### Comparison of the prognostic values of serum lipids

To compare the predictive value of lipid indicators for the outcome of patients with CRC, ROC curves were generated for the 3- and 5-year outcomes, and the AUCs were calculated. Among all lipid indicators, ApoA-I had the highest AUC for both 3- and 5-year PFS (**Figures S3A, B**). Similarly, compared with HDL, ApoA-I/ApoB, TG, TC, ApoB, LDL, and Lp(a), ApoA-I exhibited the highest predictive accuracy for 3- and 5-year OS (**Figures S3C, D**).

### Kaplan–Meier survival analysis

**Figure 1:**
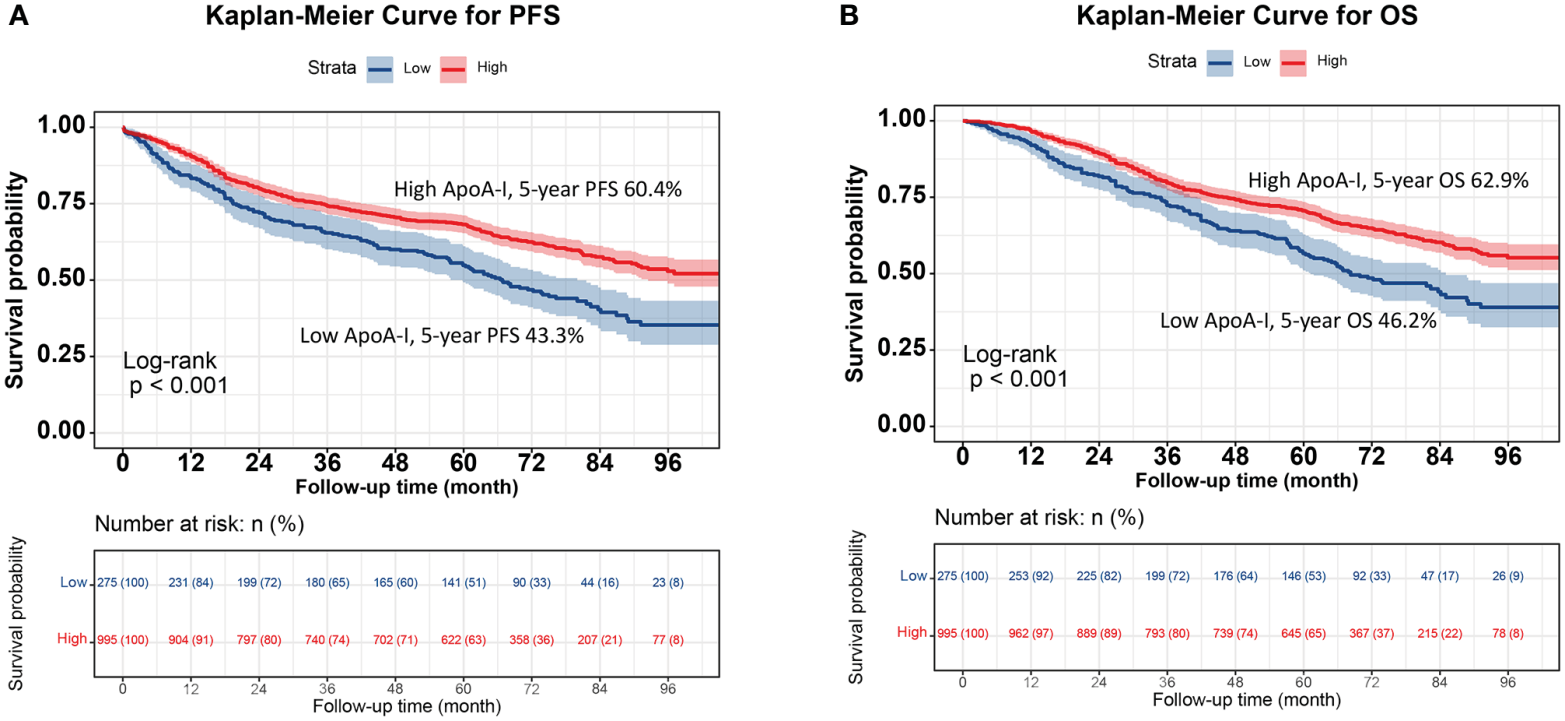
Kaplan-Meier curve of ApoA-I for PFS and OS in patients with colorectal cancer. **(A)**, Kaplan-Meier curve for PFS (High ApoA-I vs Low ApoA-I; 60.4% vs 43.3%); **(B)**, Kaplan-Meier curve for OS (High ApoA-I vs Low ApoA-I; 62.9% vs 46.2%).

**Figure 2:**
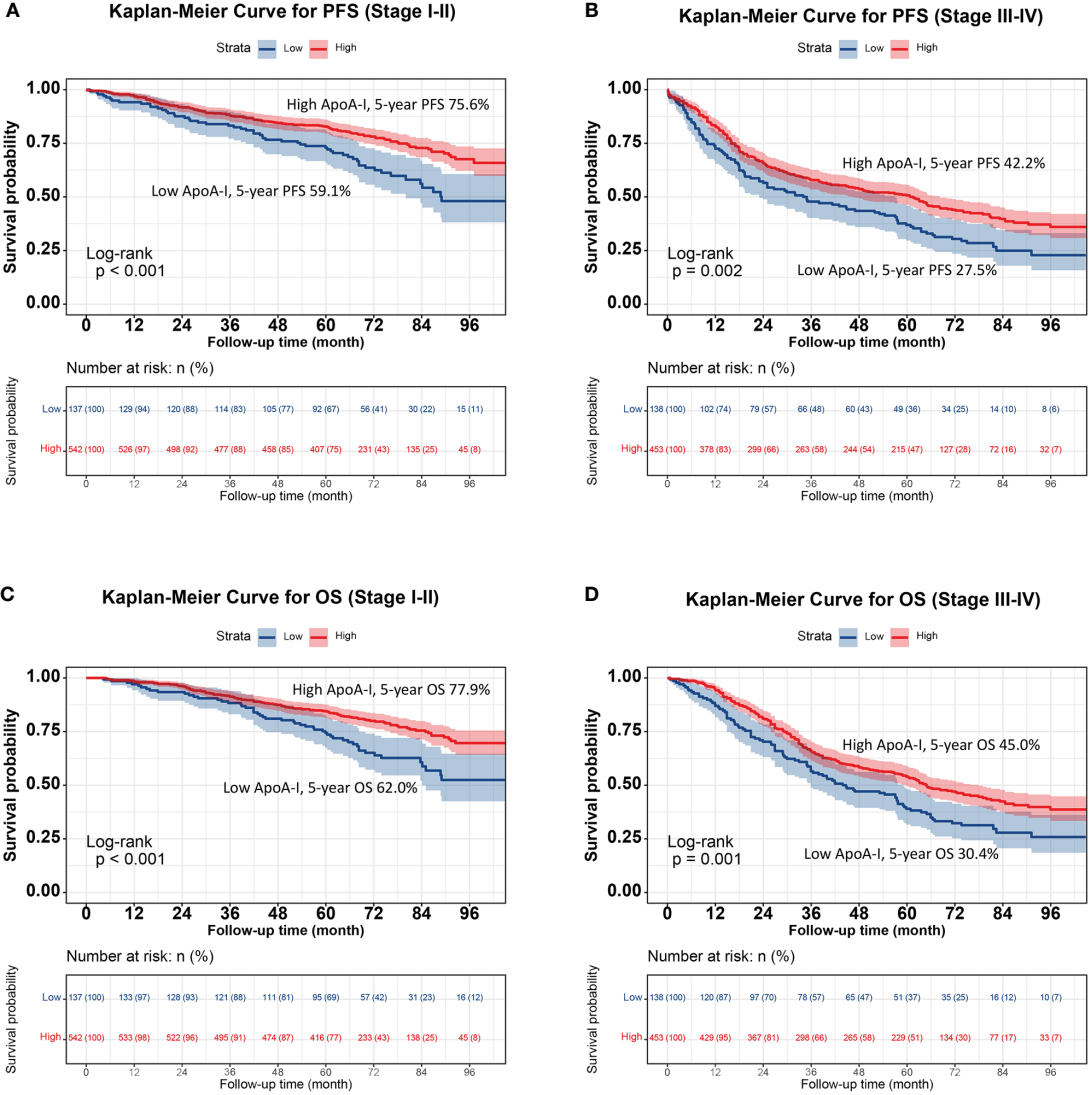
Stratified survival analysis of ApoA-I based on TNM stage subgroup. **(A)**, Kaplan-Meier curve for PFS (Stage I-II); **(B)**, Kaplan-Meier curve for OS (Stage I-II); **(C)**, Kaplan-Meier curve for OS (Stage III-IV); **(D)**, Kaplan-Meier curve for OS (Stage III-IV).

The high ApoA-I group had significantly higher survival rates compared to the low ApoA-I group at the same time points, indicating a significant association between ApoA-I levels and PFS and OS in patients with CRC (PFS: 64.8% vs. 45.2%, *P* < 0.001; OS: 66.1% vs. 48.6%, *P* < 0.001) (**Figure 1**). Considering the prognostic implications of TNM stage, tumor location, and CEA level, a subgroup analysis was performed using the K–M method among patients diagnosed with CRC. In the TNM stage subgroup analyses, the low ApoA-I group displayed worse PFS and OS than the high ApoA-I group in both stage I–II and III–IV CRC (**Figure 2**). In the tumor location subgroup analyses, the low ApoA-I group also exhibited worse PFS and OS than the high ApoA-I group (**Figures S4**, **S5**). In the CEA subgroup analyses, ApoA-I effectively differentiated between PFS and OS in patients with CRC, with better discrimination in the high CEA group than in the normal CEA group (**Figure S6**).

### Prognostic value of ApoA-I

**Figure 3:**
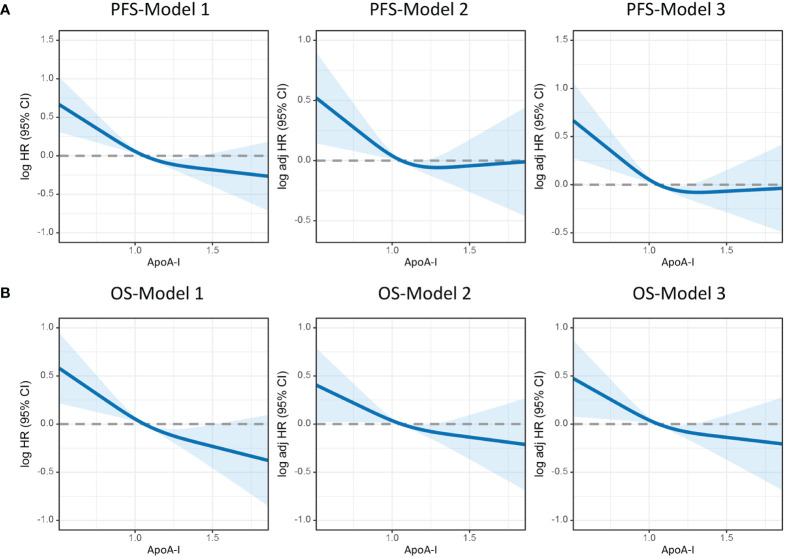
Restricted cubic spline model analysis was conducted to assess the correlation between ApoA-I levels and PFS/OS in patients with colorectal cancer. **(A)**, PFS; **(B)**, OS. Model 1: Not adjusted. Model 2: Adjusted for sex, age, and BMI. Model 3: Adjusted for sex, age, BMI, hypertension, diabetes, T stage, N stage, M stage, tumor size, perineural invasion, vascular invasion, differentiation, radiotherapy, chemotherapy.

**Figure 3** illustrates the utilization of RCS to visually depict the flexible relationship between ApoA-I levels and both PFS and OS across various adjusted models. The results indicated a gradual decrease in the hazard ratio [HR] for survival with increasing ApoA-I levels. This trend remained consistent across different models. **Tables 1** and **2** show the results of more detailed analyses of the relationships between ApoA-I levels and PFS and OS. As a continuous variable, every SD increase in ApoA-I was associated with a 12.0% decrease in PFS risk (HR 0.880; 95% confidence interval [CI], 0.801–0.968; *P* = 0.009) and an 11.2% decrease in OS risk (HR 0.888; 95%CI, 0.806–0.978; *P* = 0.015) in patients with CRC. High ApoA-I levels were associated with a 27.9% lower risk of adverse PFS (HR 0.721; 95%CI, 0.59–0.881; *P* = 0.001) and a 26.4% lower risk of adverse OS (HR 0.736; 95%CI, 0.599–0.905; *P* = 0.004) compared with low ApoA-I levels. Furthermore, quartile analysis indicated that compared with the reference level Q1 (~0.92), Q2 (0.92–1.06), Q3 (1.06–1.25), and Q4 (1.25+) were associated with decreased risks of adverse PFS and OS. In the multivariate forest plots of PFS and OS, ApoA-I was an independent protective factor in most subgroups (**Figure S7**). Additionally, examination of the relationship between ApoA-I and postoperative recurrence showed a higher risk of recurrence in the low ApoA-I group than in the high ApoA-I group (25.7% vs. 33.8%, *P* = 0.010). Multivariate logistic regression analysis revealed that for every SD increase in ApoA-I, the risk of disease recurrence decreased by 54% (odds ratio [OR], 0.460; 95%CI, 0.250–0.830; *P* = 0.010). Compared with the high ApoA-I group (≥0.9 g/L), the low ApoA-I group (<0.9 g/L) had a 32.5% higher risk of disease recurrence (OR, 0.675; 95%CI, 0.481–0.946; *P* = 0.0225) (**Table 3**).

**Table 1:** Association between ApoA-I and PFS of patients with colorectal cancer.

ApoA-I	Model 1	p value	Model 2	p value	Model 3	p value
Continuous (per SD)	0.837 (0.768,0.913)	<0.001	0.905 (0.826,0.992)	0.032	0.88 (0.801,0.968)	0.009
Cutoff value (High)	0.629 (0.522,0.757)	<0.001	0.75 (0.616,0.912)	0.004	0.721 (0.59,0.881)	0.001
Quartiles						
Q1 (~0.92)	ref		ref		ref	
Q2 (0.92~1.06)	0.612 (0.484,0.773)	<0.001	0.669 (0.527,0.848)	0.001	0.637 (0.5,0.812)	<0.001
Q3 (1.06~1.25)	0.757 (0.607,0.944)	0.013	0.84 (0.669,1.055)	0.133	0.783 (0.621,0.988)	0.04
Q4 (1.25~)	0.627 (0.495,0.795)	<0.001	0.785 (0.609,1.01)	0.06	0.736 (0.567,0.955)	0.021
p for trend		0.001		0.168		0.071

Model 1: Not adjusted.

Model 2: Adjusted for sex, age, and BMI.

Model 3: Adjusted for sex, age, BMI, hypertension, diabetes, T stage, N stage, M stage, tumor size, perineural invasion, vascular invasion, differentiation, radiotherapy, chemotherapy.

**Table 2:** Association between ApoA-I and OS of patients with colorectal cancer.

ApoA-I	Model 1	p value	Model 2	p value	Model 3	p value
Continuous (per SD)	0.836 (0.765,0.913)	<0.001	0.896 (0.818,0.983)	0.02	0.888 (0.806,0.978)	0.015
Cutoff value (High)	0.622 (0.514,0.752)	<0.001	0.74 (0.606,0.904)	0.003	0.736 (0.599,0.905)	0.004
Quartiles						
Q1 (~0.92)	ref		ref		ref	
Q2 (0.92~1.06)	0.616 (0.485,0.783)	<0.001	0.69 (0.541,0.88)	0.003	0.677 (0.529,0.868)	0.002
Q3 (1.06~1.25)	0.742 (0.591,0.932)	0.01	0.813 (0.643,1.027)	0.083	0.782 (0.615,0.993)	0.044
Q4 (1.25~)	0.611 (0.478,0.78)	<0.001	0.743 (0.574,0.962)	0.024	0.732 (0.559,0.958)	0.023
p for trend		0.001		0.059		0.051

Model 1: Not adjusted.

Model 2: Adjusted for sex, age, and BMI.

Model 3: Adjusted for sex, age, BMI, hypertension, diabetes, T stage, N stage, M stage, tumor size, perineural invasion, vascular invasion, differentiation, radiotherapy, chemotherapy.

**Table 3:** Association between ApoA-I and recurrence of patients with colorectal cancer.

ApoA-I	Model 1	p value	Model 2	p value	Model 3	p value
Continuous (per SD)	0.487 (0.297,0.798)	0.0043	0.619 (0.354,1.082)	0.0921	0.46 (0.25,0.83)	0.01
Cutoff value (High)	0.678 (0.509,0.904)	0.008	0.678 (0.509,0.904)	0.008	0.675 (0.481,0.946)	0.0225
Quartiles						
Q1 (~0.92)	ref		ref		ref	
Q2 (0.92~1.06)	0.714 (0.505,1.007)	0.055	0.725(0.494,1.065)	0.101	0.668(0.451,0.989)	0.044
Q3 (1.06~1.25)	0.813 (0.579,1.141)	0.231	0.865(0.592,1.264)	0.455	0.757(0.511,1.123)	0.166
Q4 (1.25~)	0.67 (0.473,0.948)	0.024	0.825(0.553,1.229)	0.344	0.685(0.452,1.037)	0.074
p for trend		0.541		0.658		0.365

Model 1: Not adjusted.

Model 2: Adjusted for sex, age, and BMI.

Model 3: Adjusted for sex, age, BMI, hypertension, diabetes, T stage, N stage, M stage, tumor size, perineural invasion, vascular invasion, differentiation, radiotherapy, chemotherapy.

### ApoA-I-based prediction nomograms

**Figure 4:**
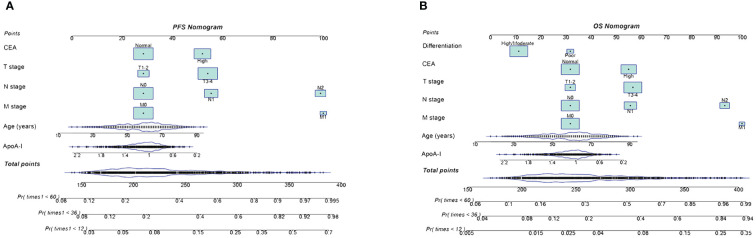
Construction the PFS and OS nomograms in CRC patients. **(A)**, PFS nomogram; **(B)**, OS nomogram.These nomograms comprise specific clinical features, with each feature corresponding to a specific point. A straight line can be drawn through these points on the axis to calculate the score for each feature. After summing the scores of these features, positioned on the total point axis, the probability of risk can be calculated by drawing downward to the prediction axis. Based on the results of Cox proportional hazards regression models, we developed these nomograms to predict the PFS/OS in CRC patients.

Preoperative ApoA-I, age, T stage, N stage, M stage, and CEA independently predicted both PFS and OS (**Tables S2**, **S3**). Consequently, nomograms utilizing these indicators were created to forecast 1–5-year PFS and OS for patients diagnosed with CRC (**Figures 4A, B**). The C-index of the nomograms indicated their good predictive performance for OS (0.726) and PFS (0.719) in patients with CRC. Further ROC analysis demonstrated the high predictive performance of these nomograms for PFS and OS, with 1-, 3-, and 5-year AUC values of (0.799 vs. 0.776 vs. 0.764) and (0.767 vs. 0.773 vs. 0.766), respectively (**Figure S8**). The calibration curves exhibited satisfactory concordance between the predicted values and actual clinical outcomes (**Figure S9**). Moreover, DCA to compare the clinical benefits of ApoA-I-based nomograms with those of traditional tumor staging showed superior clinical benefits of the ApoA-I-based nomograms for both PFS and OS, (**Figure S10A, B**). Subsequently, based on the median scores of the nomograms, the patients were divided into high-score and low-score groups. The high-scoring group showed significantly worse PFS and OS compared to the low-scoring group (**Figure S11A, B**).

## Discussion

Mounting evidence suggests the critical role of systemic inflammation in both cancer onset and progression (23, 24). Serum markers of systemic inflammation, such as serum C-reactive protein and procalcitonin, have been identified as adverse prognostic factors in various cancers (25, 26). Interestingly, serum ApoA-I levels have shown a significant association with systemic inflammatory markers. In their study involving 144 patients with CRC, Sirnio et al. (26) observed a robust negative relationship between serum ApoA-I levels and systemic inflammatory markers, including serum C-reactive protein level, interleukin-8 level, and blood neutrophil count. Furthermore, the authors reported a notable association between low serum ApoA-I levels and advanced T and TNM stages. Consequently, serum ApoA-I levels show promise as an indicator of both systemic inflammation and tumor progression. Similarly, the results of the present study revealed a significant association between ApoA-I levels and tumor progression. Low ApoA-I levels were significantly correlated with advanced pathological staging, larger tumor diameters, and higher CEA levels. Additionally, compared with patients with high ApoA-I levels, patients with low ApoA-I levels exhibited higher rates of recurrence, mortality, and hospitalization burden. Thus, decreased ApoA-I levels may serve as an indicator of aggressive tumor behavior and poor prognosis. The correlation between serum ApoA-I levels and tumor characteristics, as revealed in this study, can provide tailored guidance for treatment decisions and prognosis assessment.

ApoA-I itself possesses antitumor properties by reducing angiogenesis, altering immune cells, enhancing cholesterol efflux, and reversing sterol transport in cancer cells, which may inhibit tumor cell proliferation or growth (21). Although ApoA-I is linked to the development of various tumors and is a potential biochemical marker for diagnosing various cancers (27, 28), limited research has explored the correlation between ApoA-I level and the outcomes of patients with CRC. A study by Quan et al. (19) involving 508 participants, suggested that ApoA-I could serve as a prognostic factor in patients with metastatic CRC treated with bevacizumab. Zhang et al.’s meta-analysis found that serum ApoA-I could serve as a non-invasive marker for predicting the prognosis of various tumors, including CRC (29). Sirniö et al. also identified serum ApoA-I as a promising additional prognostic parameter for CRC (26). However, these studies were limited by small sample sizes or focused mainly on late-stage patients. Gu et al. evaluated four key blood lipid factors and established a novel lipoprotein cholesterol-apolipoprotein score for predicting the prognosis of patients undergoing CRC resection (30). In our study, we compared the prognostic values of eight common blood lipid factors and found that ApoA-I was the optimal blood lipid factor for predicting PFS and OS in CRC patients. This provides valuable insight for the clinical use of blood lipid factors in assessing the prognosis of CRC patients. Furthermore, unlike previous studies, we extensively explored the relationship between ApoA-I and PFS, OS, and recurrence in CRC patients. The results of the present study revealed higher PFS and OS rates in the low ApoA-I group compared to those in the high ApoA-I group, with improvements of 17.1% and 16.7%, respectively. Multivariate analysis indicated that ApoA-I was an independent predictor of PFS and OS in patients with CRC, with HR values of 0.778 and 0.776, respectively, irrespective of the TNM stage. Moreover, ApoA-I was an independent predictive factor for disease recurrence in patients with CRC. These findings suggest that ApoA-I may be a valuable prognostic indicator for individuals diagnosed with CRC and offer new insights into previous literature.

Despite the widespread utilization of TNM staging as the primary tool for treatment decisions and prognosis evaluation in patients with CRC, it is important to note that patient outcomes can vary significantly even within the same pathological stage. Therefore, identifying effective prognostic tools that complement TNM staging is crucial. Within the TNM subgroups, ApoA-I level effectively differentiated between patient outcomes in both stage I–II and III–IV CRC. This finding underscores the utility of ApoA-I as a valuable adjunct to TNM staging for prognostic assessments. Additionally, ApoA-I level was an effective prognostic predictor in both colon and rectal cancers. Within the CEA subgroups, ApoA-I effectively distinguished patient outcomes in each subgroup, with stronger prognostic discrimination in the high CEA subgroup than in the normal CEA subgroup. Collectively, these findings suggest that serum ApoA-I level may be a valuable indicator for predicting the outcome of patients with CRC.

While the current prognostic classification for CRC primarily relies on TNM stage, the predictive value of a single parameter is limited. Therefore, comprehensively integrating multiple indicators are essential for accurate prognostic prediction in patients with CRC. In the present study, besides ApoA-I, patient age and T stage, N stage, M stage, and CEA level were identified as independent influencing factors in patients with CRC. Incorporating various effective prognostic parameters is invaluable when evaluating the outcome of patients with CRC. Thus, these variables were integrated to construct ApoA-I-based prediction nomograms. The C-index and calibration curves indicated that the ApoA-I-based nomograms exhibited excellent predictive accuracy. Furthermore, compared with the traditional TNM staging system, the ApoA-I-based nomograms offered superior clinical benefits. These findings suggest that ApoA-I-based nomograms incorporating a range of prognostic parameters are effective tools for predicting the outcome of patients with CRC. These tools can provide personalized assistance for clinical decision-making in the care of patients with CRC.

### Study strengths

Through its analysis of a large clinical cohort, the results of this support serum ApoA-I levels as a promising biomarker for predicting the prognosis of patients with CRC. Multiple study outcomes, including PFS, OS, and disease recurrence, were incorporated to explore the prognostic value of serum ApoA-I levels in patients with CRC. Among all serum lipid markers, ApoA-I exhibited the highest predictive accuracy for 3- and 5-year PFS/OS and OS. This finding highlights the significance of ApoA-I as the most representative indicator of lipid metabolism in predicting the outcome of patients with CRC. Furthermore, serum ApoA-I levels can serve as useful prognostic complements to TNM staging and CEA markers. By integrating prognostic variables, predictive nomograms based on ApoA-I were developed and their excellent prognostic prediction efficacy was confirmed. These findings demonstrated the strength of this study.

### Limitations

This study has several limitations. Firstly, the retrospective single-center design made it difficult to completely avoid potential selection bias. Secondly, the study population consisted solely of Chinese individuals. Further exploration is required to determine the applicability of these findings to other populations. Finally, the ApoA-I-based nomograms developed in this study require extensive external validation in diverse populations before they can be applied in clinical practice.

## Conclusion

Decreased serum ApoA-I levels are associated with stronger tumor invasiveness, greater disease burden, and poorer prognosis. Serum ApoA-I levels are an independent factor affecting the prognosis of patients and are a useful supplement to TNM staging. The ApoA-I-based nomograms are effective tools for the comprehensive assessment of prognosis in patients with CRC. These findings have the potential to offer personalized guidance for treatment decisions and prognosis assessment.

## Data availability statement

The original contributions presented in the study are included in the article/**Supplementary Material**. Further inquiries can be directed to the corresponding authors.

## Ethics statement

The studies involving humans were approved by The First Affiliated Hospital, Guangxi Medical University (registration number: NO.2022-KY-(043)). The studies were conducted in accordance with the local legislation and institutional requirements. The participants provided their written informed consent to participate in this study.

## Author contributions

HX: Data curation, Investigation, Software, Writing – original draft, Writing – review & editing. LW: Data curation, Formal analysis, Methodology, Supervision, Writing – review & editing. QW: Formal analysis, Project administration, Validation, Writing – review & editing. ST: Funding acquisition, Resources, Visualization, Writing – review & editing. JG: Conceptualization, Writing – review & editing.
