# Supplementary material for: Apolipoprotein A-I levels in the survival of patients with colorectal cancer: a retrospective study
Source: Front Endocrinol (Lausanne). 2024 Jun 11;15:1318416. doi: 10.3389/fendo.2024.1318416 (PMC11196595; doi:10.3389/fendo.2024.1318416)
Supplement: Supplementary file 1 [file DataSheet_1.docx]

**Supplementary materials**

**Figure S1.** The optimal threshold cutoff of ApoA-I using maximally selected rank statistics.

**
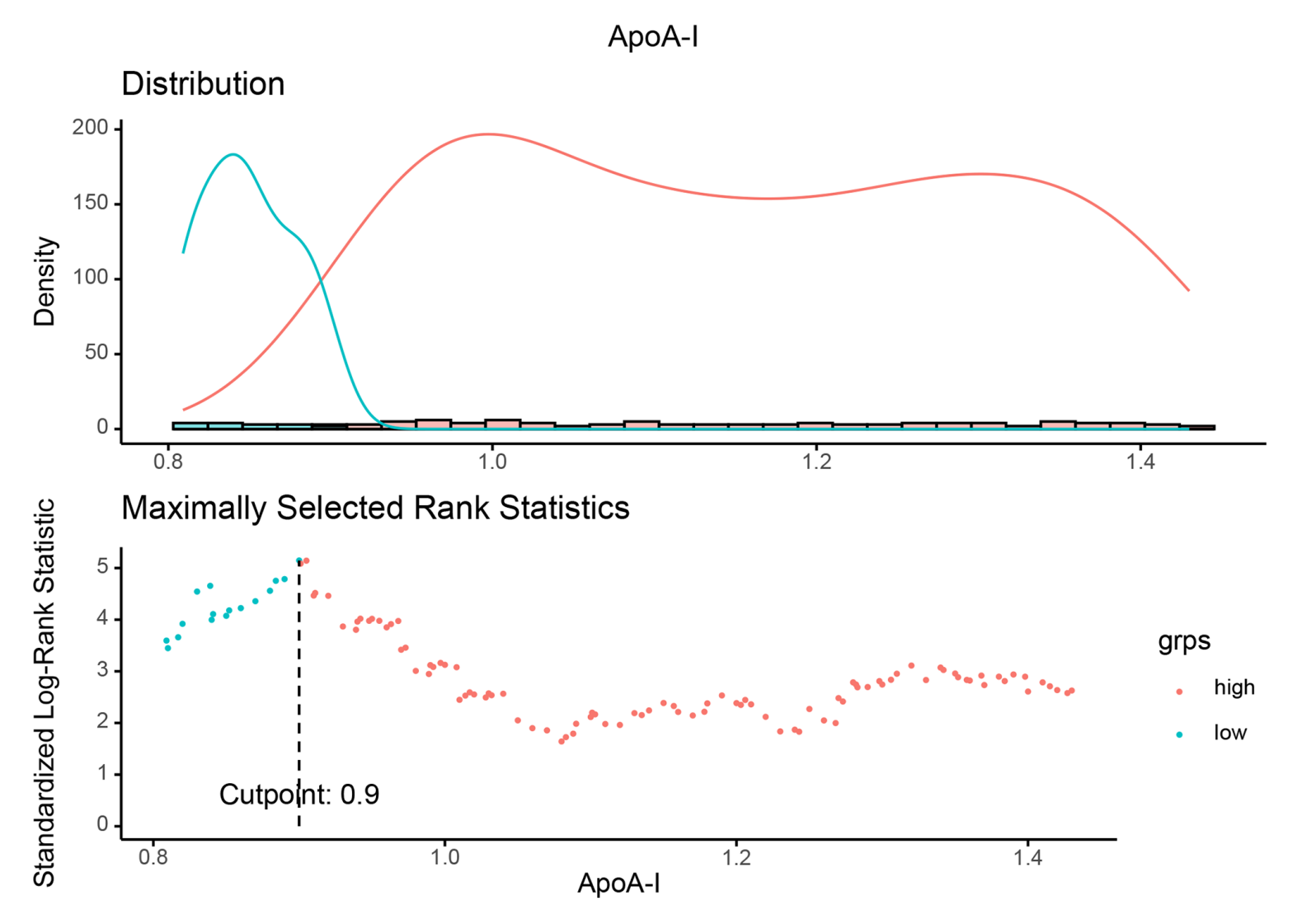
**

**Notes:** The optimal threshold for ApoA-I was determined using maximally selected rank statistics. Maximally selected rank statistics involve partitioning each value of ApoA-I separately, where each partition divides the data into two groups and simultaneously calculates a standardized statistic. This standardized statistic varies depending on the type of dependent variable but generally reflects the difference between the two groups after partitioning by a certain value. After all partitions, multiple standardized statistics are obtained, and among them, the maximum one is identified. The corresponding value of this maximum statistic is considered the optimal cut-off value.

**Figure S2.** The expression levels of ApoA-I across different clinicopathological characteristic subgroups.

**
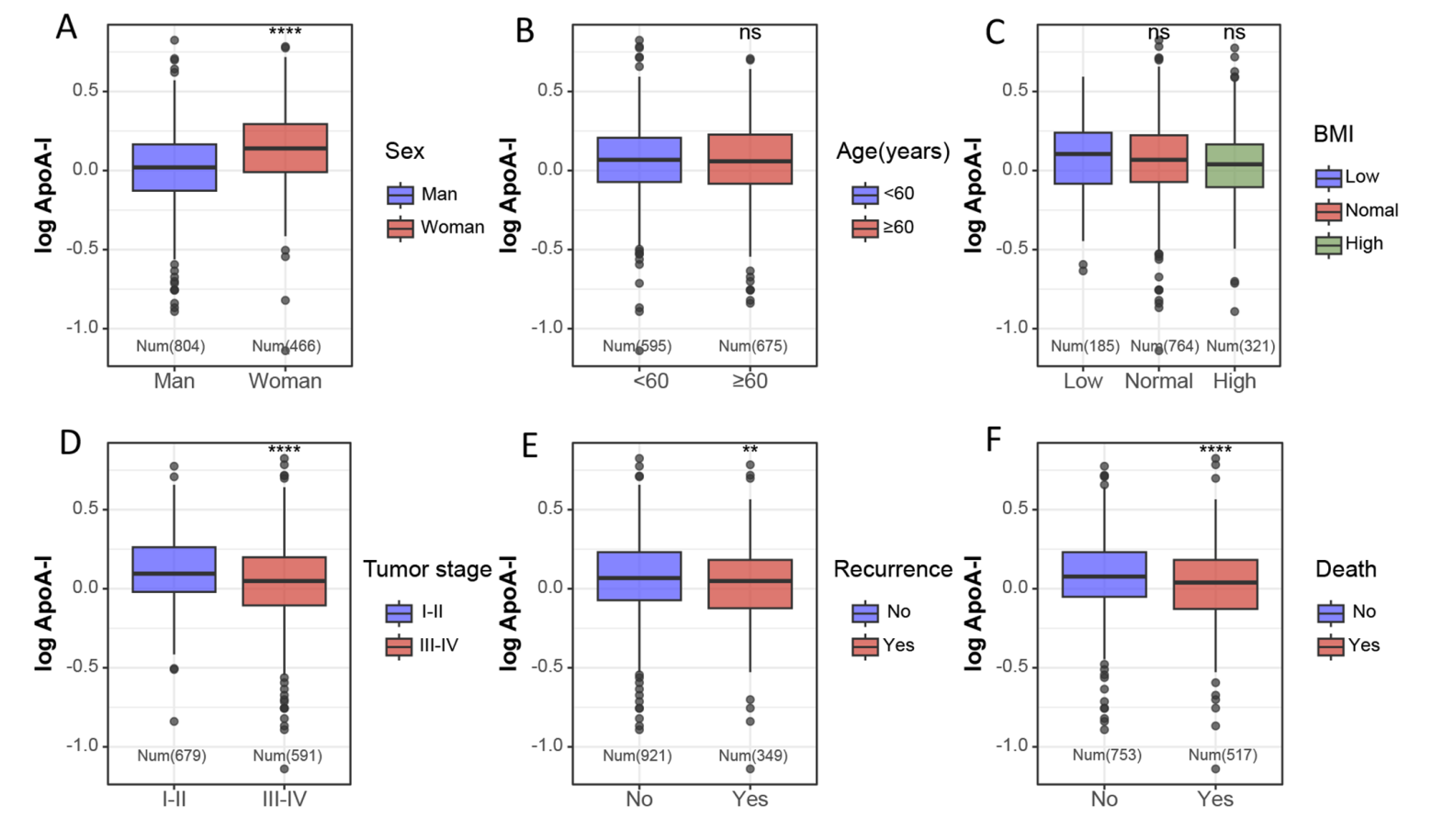
**

**Notes:** A, Sex (Man vs Woman) ; B, Age (<60 vs ≥60); C, BMI (Low vs Normal vs High); D, Tumor stage (I-II vs III-IV); E, Recurrence (No vs Yes); F, Death (No vs Yes).

**Figure S3.** Comparison of the prognostic value of blood lipid factors by ROC curve.

**
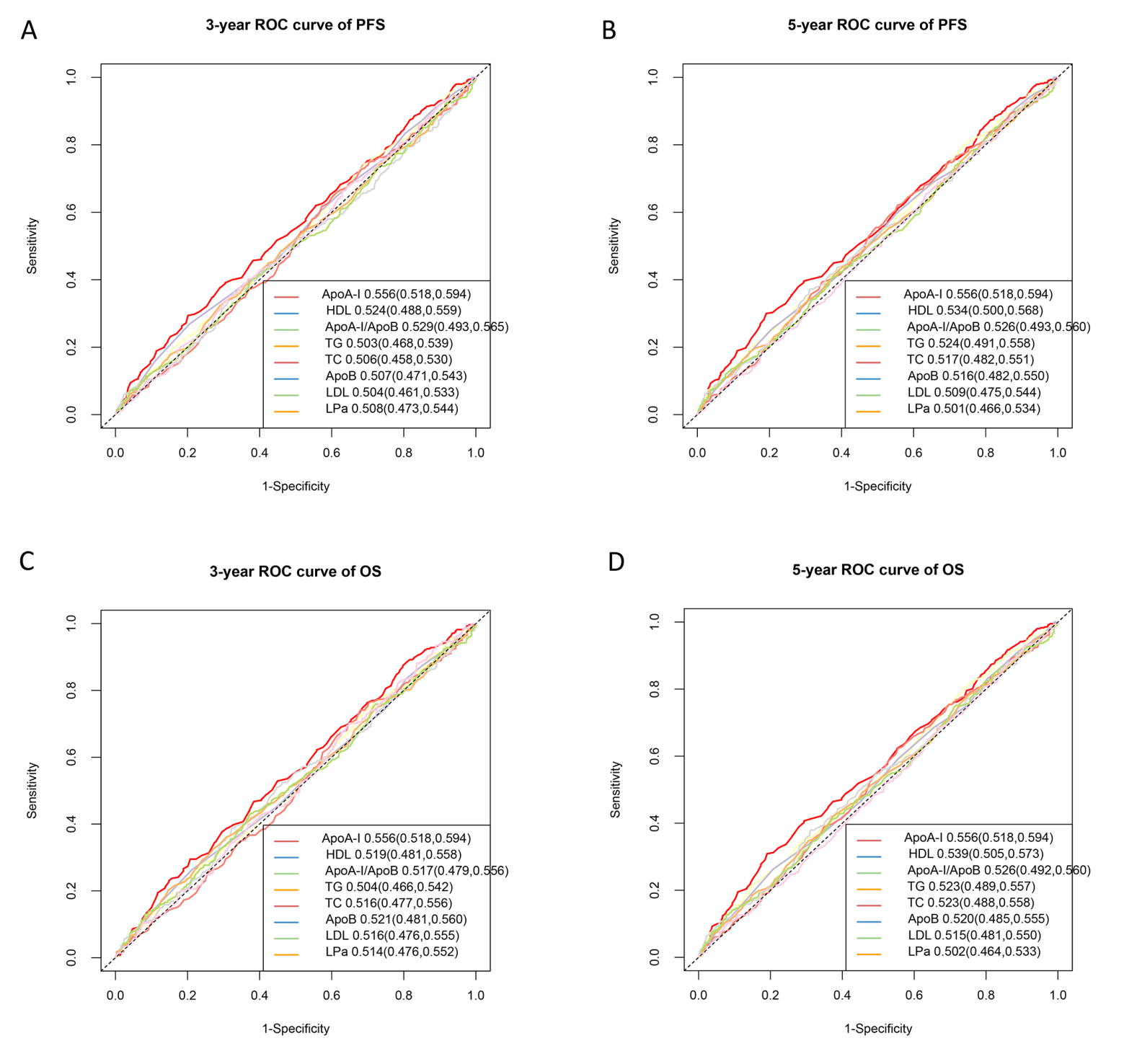
**

**Notes:** A, 3-year ROC of PFS; B, 5-year ROC of PFS; C, 3-year ROC of OS; C, 5-year ROC of OS.

**Figure S4.** Kaplan-Meier curve of ApoA-I of patients with colon cancer.


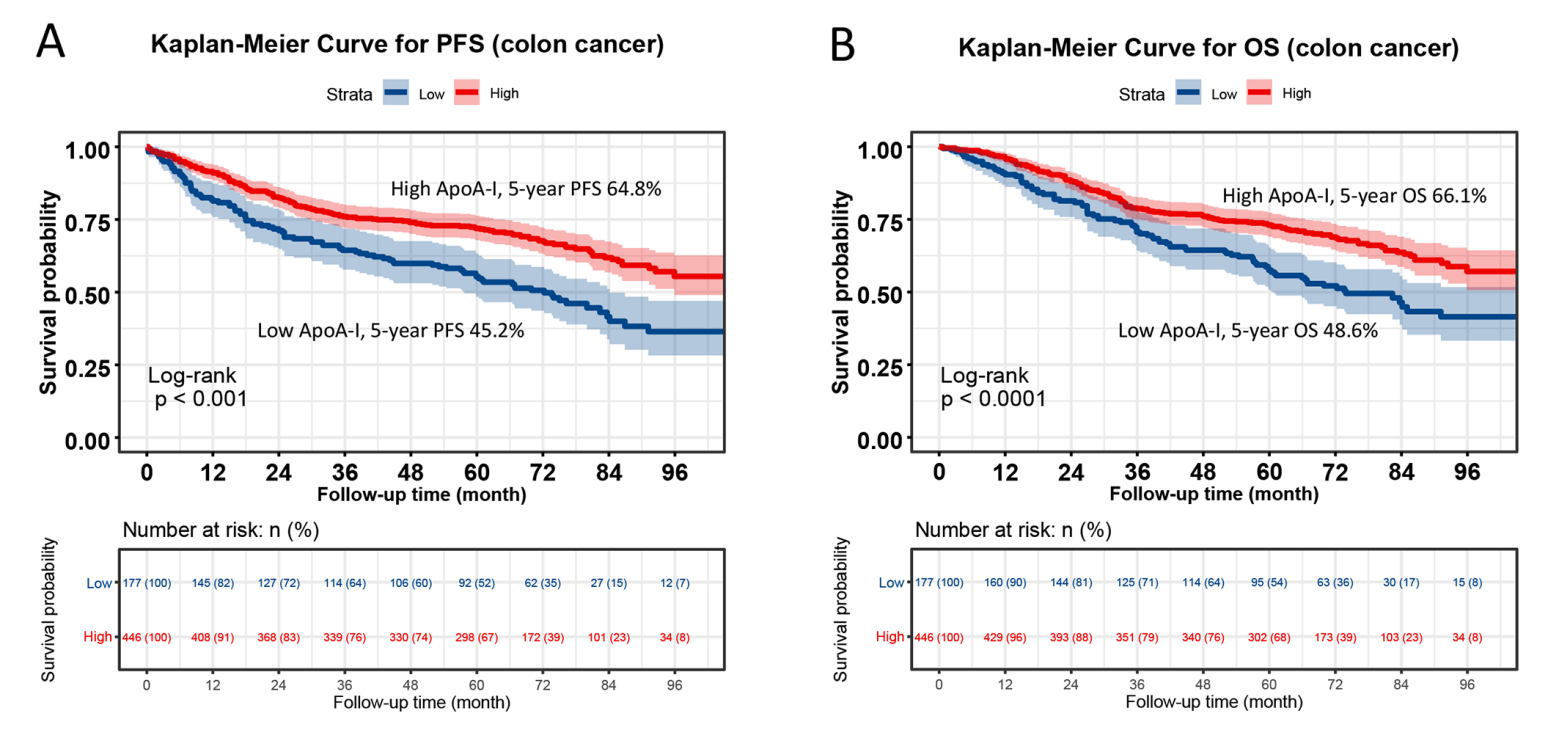


**Notes:** A, Kaplan-Meier curve of PFS (High ApoA-I vs Low ApoA-I; 64.8% vs 45.2%); B, Kaplan-Meier curve of OS (High ApoA-I vs Low ApoA-I; 66.1% vs 48.6%).

**Figure S5.** Survival curve of ApoA-I of patients with rectal cancer.


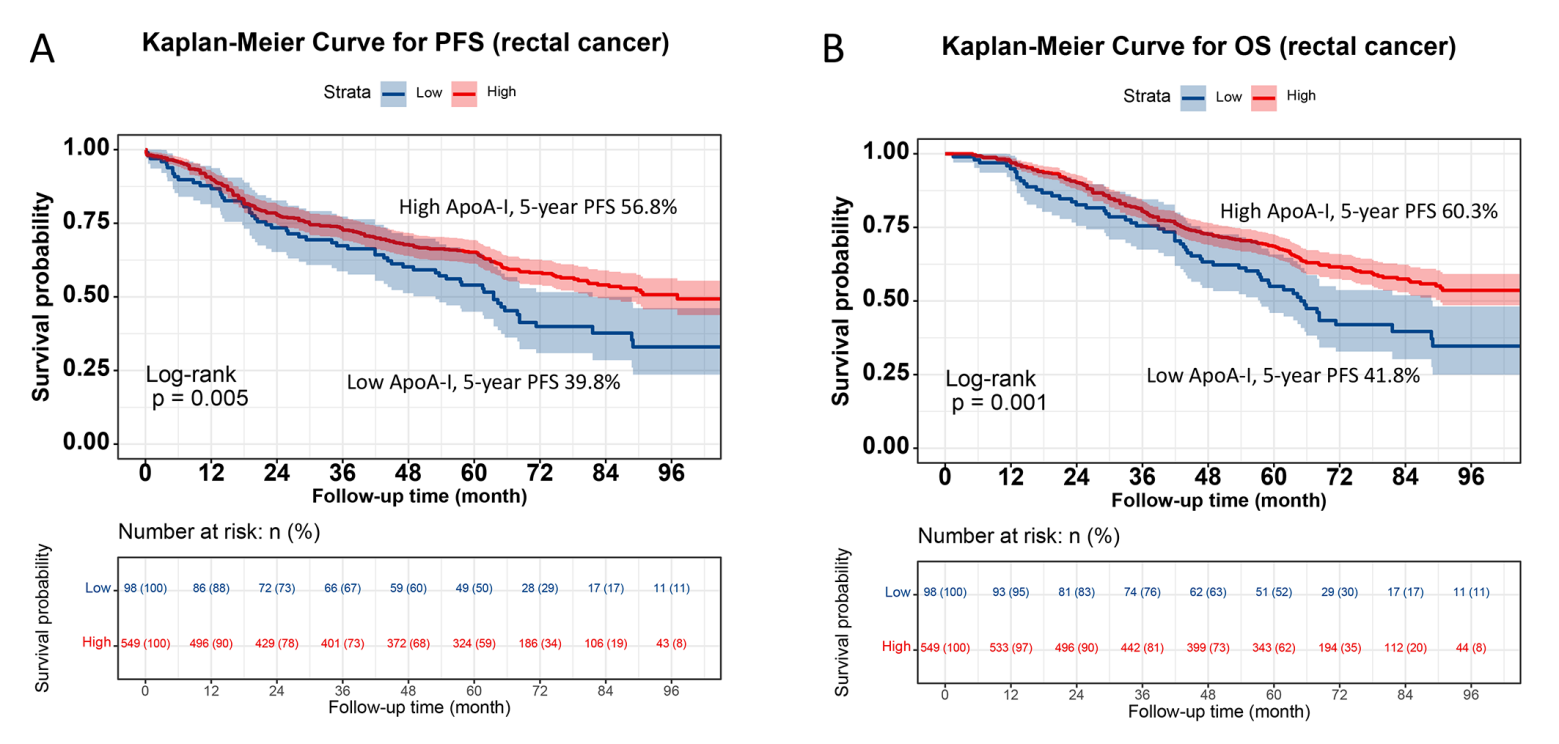


**Notes:** A, Kaplan-Meier curve of PFS (High ApoA-I vs Low ApoA-I; 56.8% vs 39.8%); B, Kaplan-Meier curve of OS (High ApoA-I vs Low ApoA-I; 60.3% vs 41.8%).

**Figure S6.** Stratified Kaplan-Meier curve of ApoA-I based on CEA subgroup.


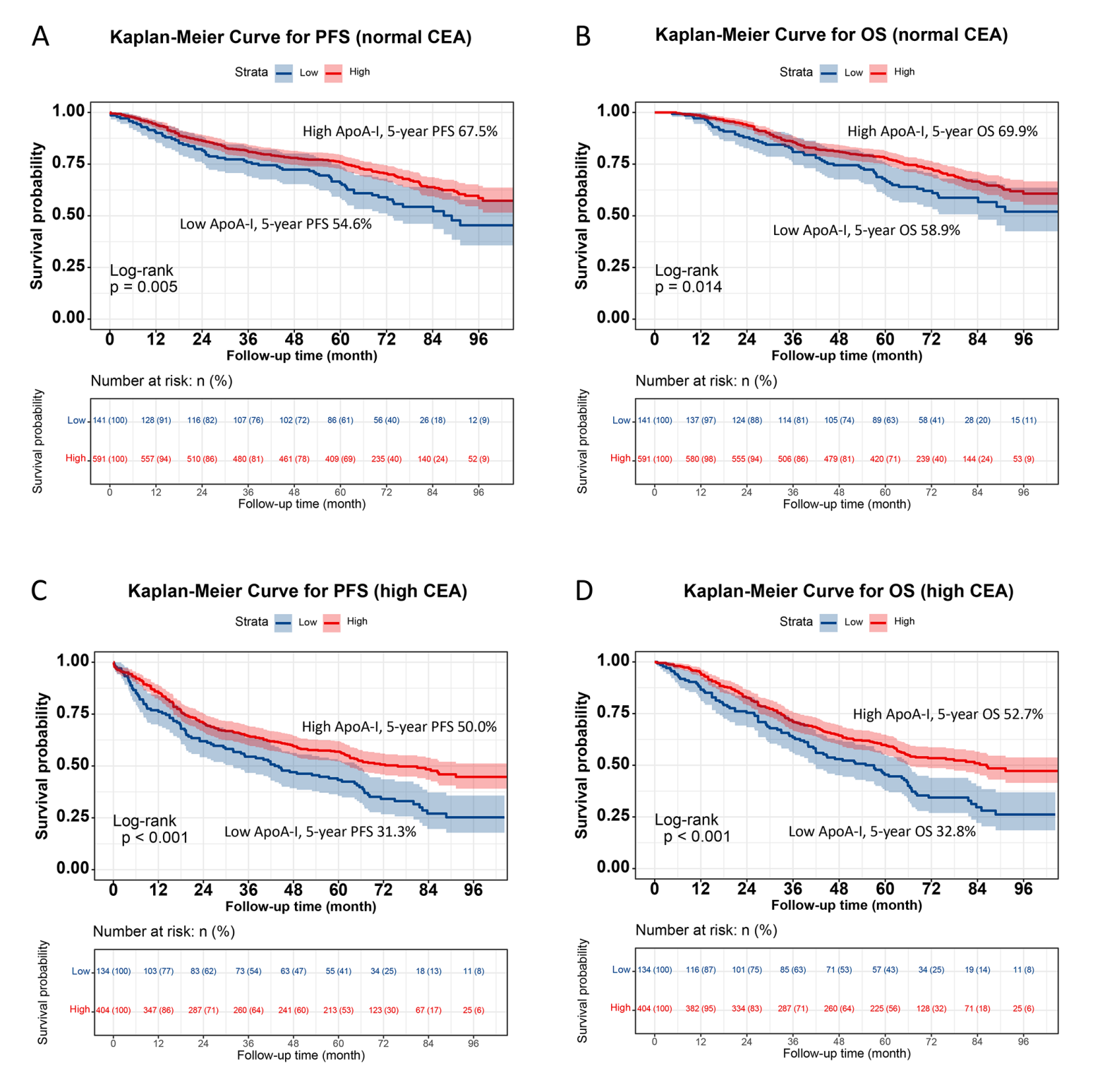


**Notes:** A, Kaplan-Meier curve of PFS at normal CEA (High ApoA-I vs Low ApoA-I; 67.5% vs 54.6%); B, Kaplan-Meier curve of OS at normal CEA (High ApoA-I vs Low ApoA-I; 69.9% vs 58.9%); C, Kaplan-Meier curve of PFS at high CEA (High ApoA-I vs Low ApoA-I; 50.0% vs 31.3%); C, Kaplan-Meier curve of PFS at high CEA(High ApoA-I vs Low ApoA-I; 52.7% vs 32.8%).

**Figure S7.** Forest plots depicting the hazard ratio of progression-free survival (A) or overall survival (B) for ApoA-I across various subgroups.


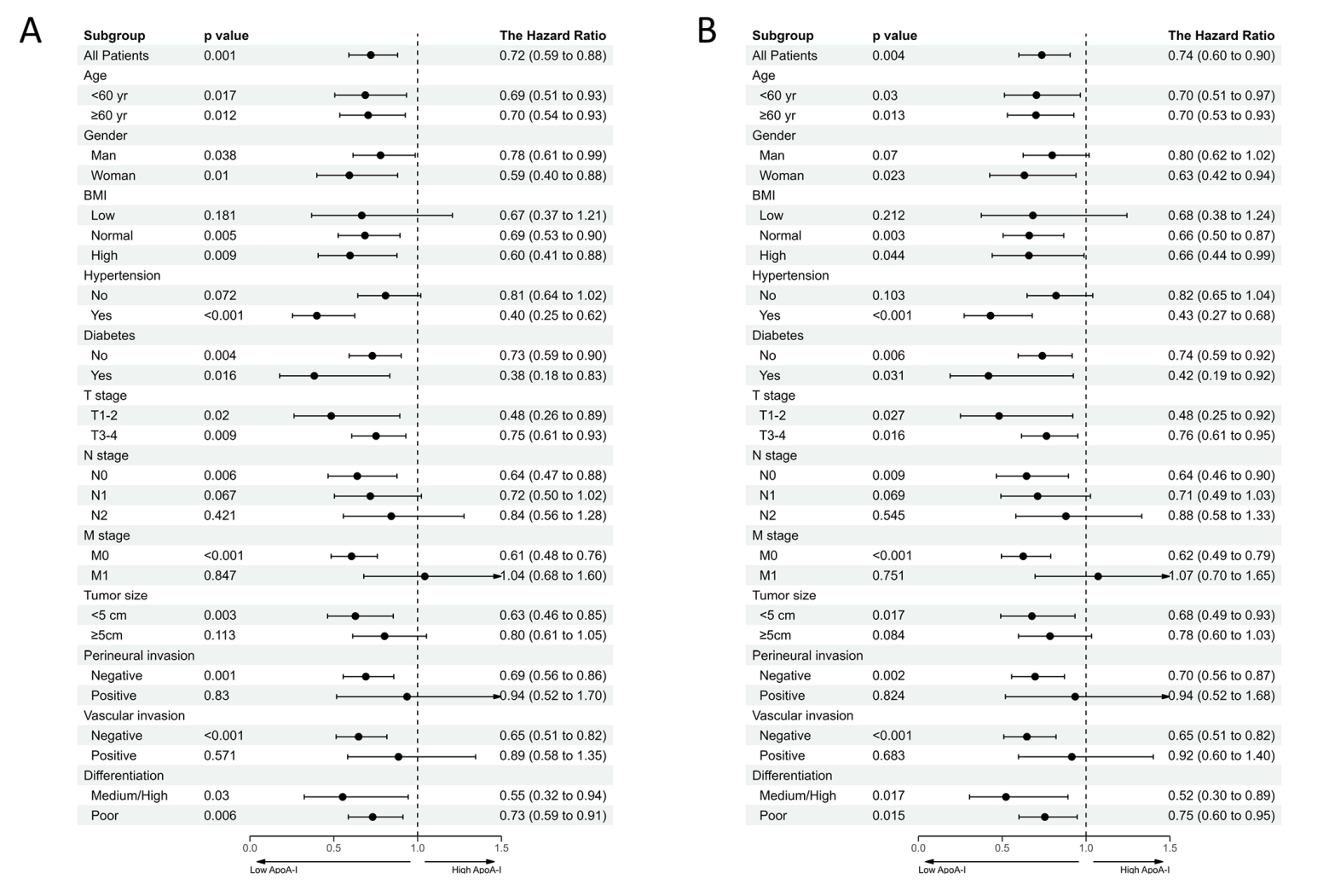


**Notes:** Adjusted for sex, age, BMI, hypertension, diabetes, T stage, N stage, M stage, tumor size, perineural invasion, vascular invasion, differentiation, radiotherapy, chemotherapy.

**Figure S8.** The 1-, 3-, and 5-year ROC of PFS and OS nomograms.

**
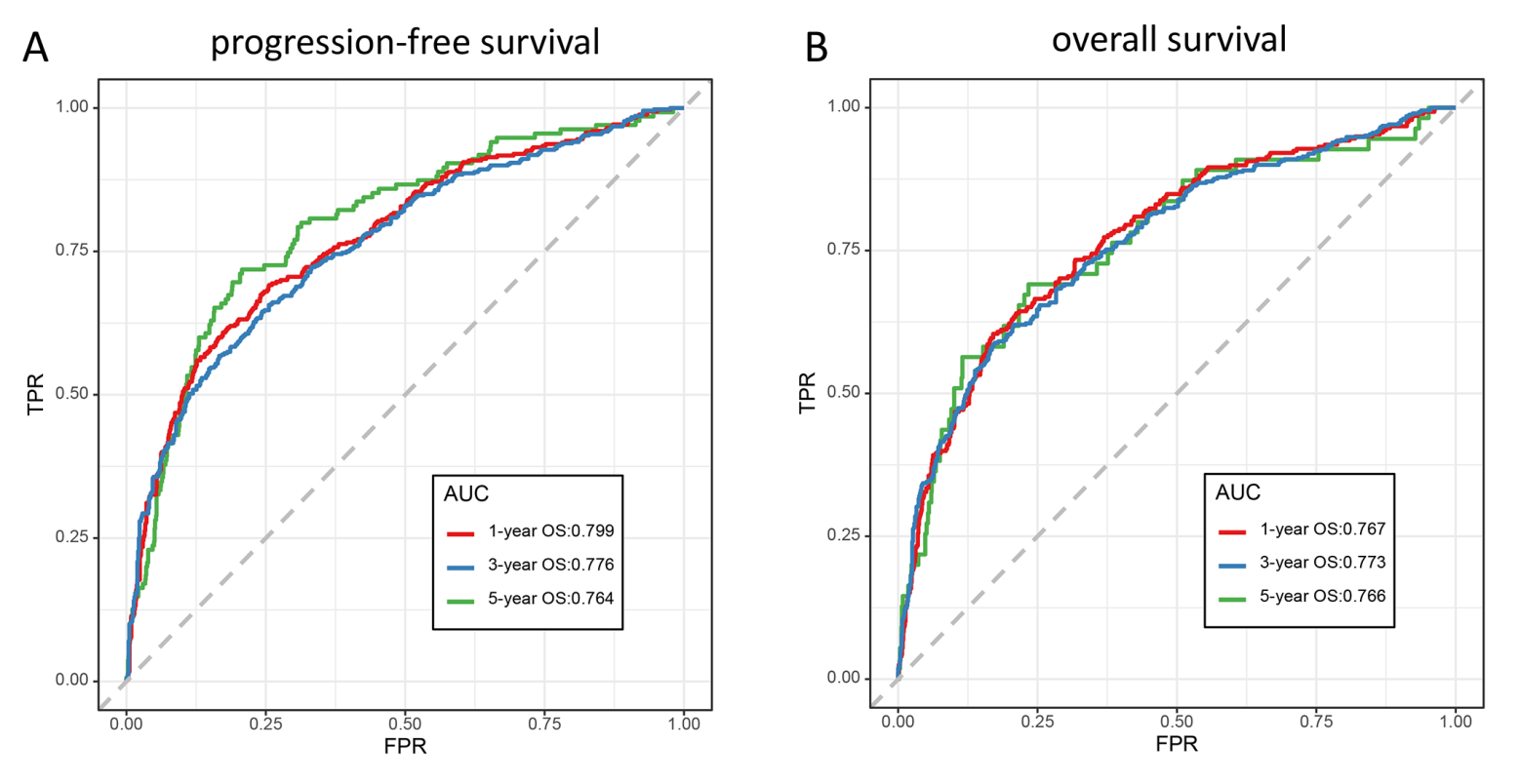
**

**Notes:** A, PFS; B, OS.

**Figure S9.** The 1-, 3-, and 5-year calibration curve of the PFS/OS nomograms.

**
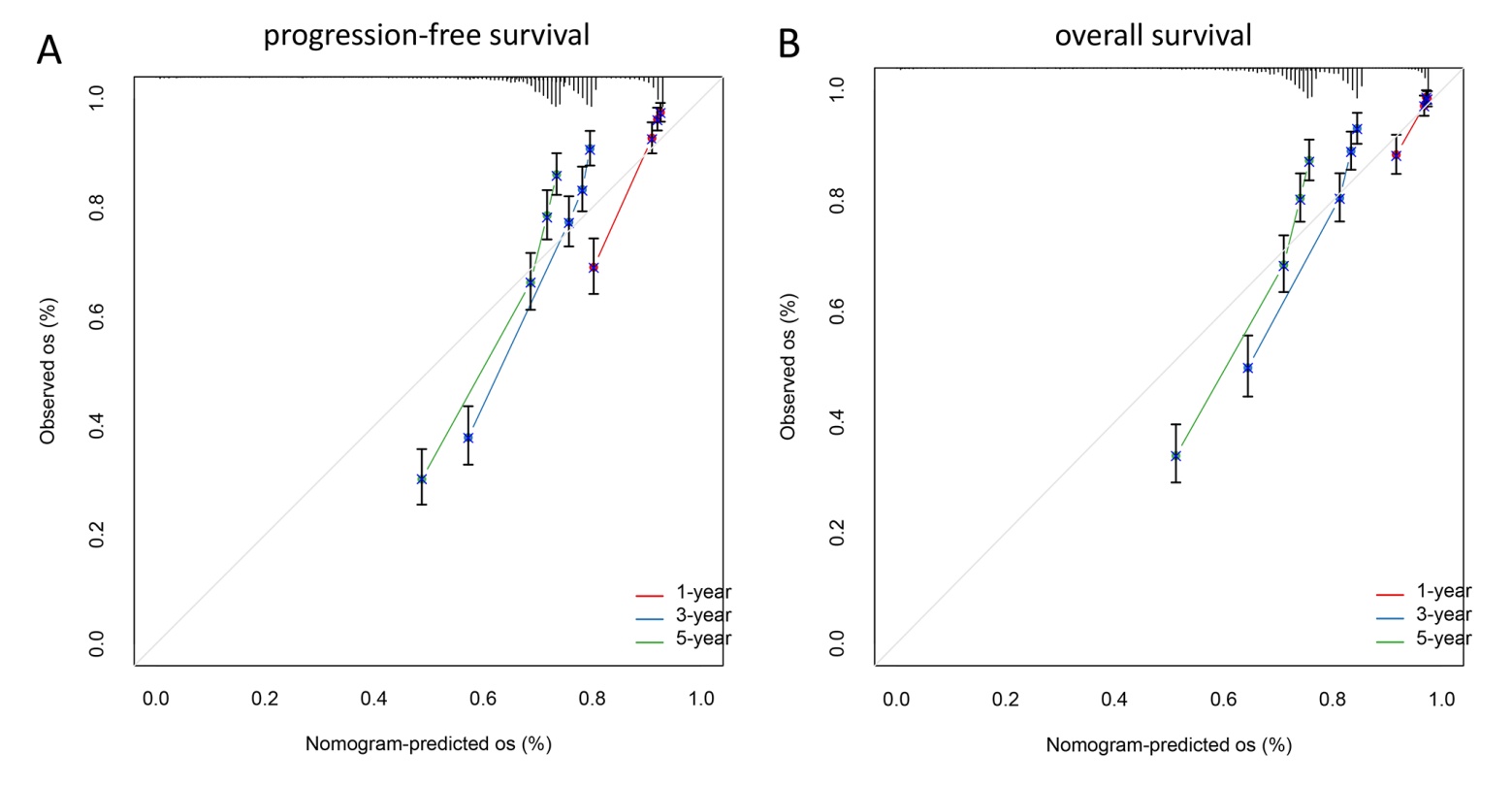
**

**Notes:** A, PFS; B, OS.

**Figure S10.** The Decision Curve Analysis of PFS/OS nomograms.

**
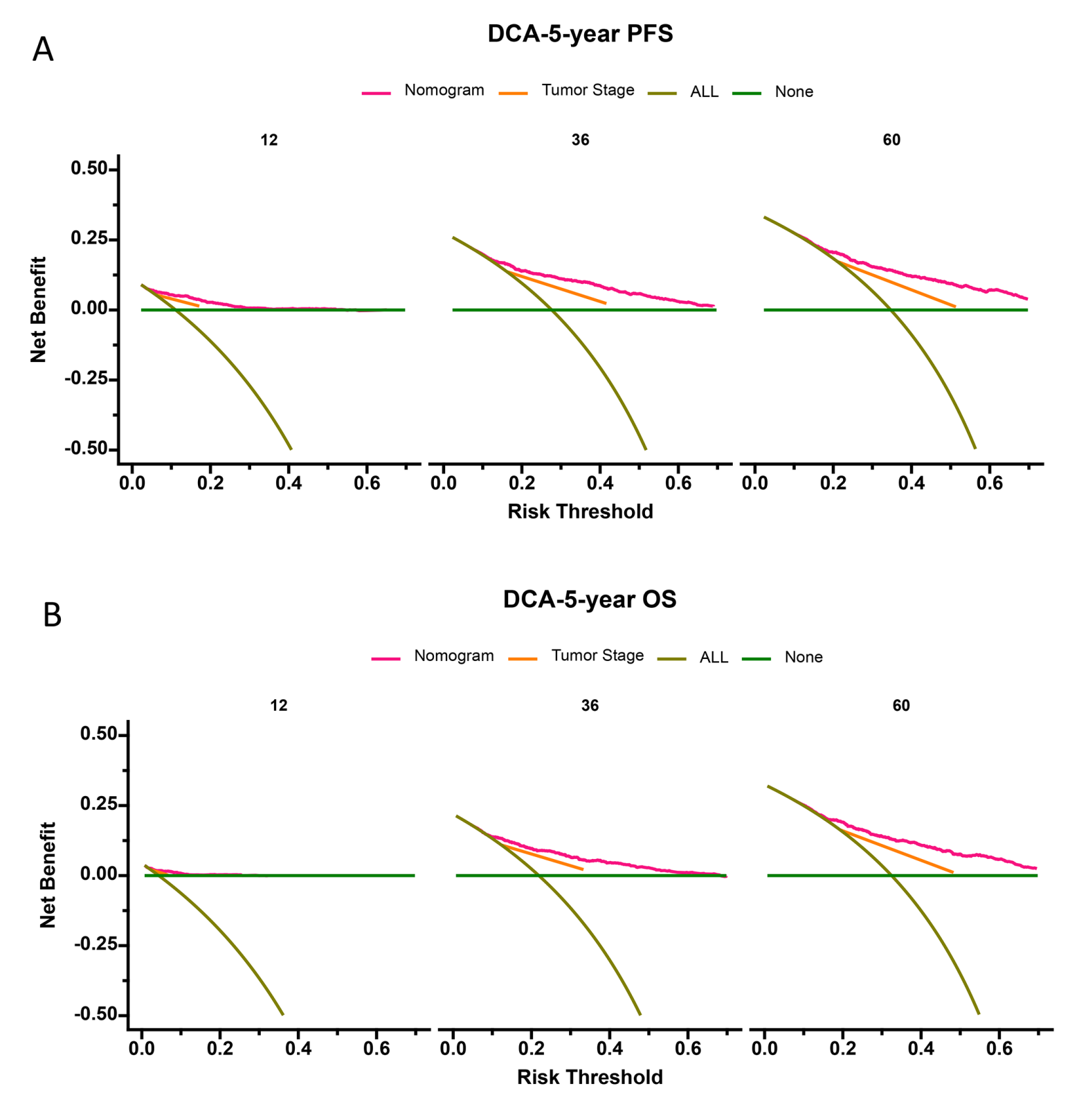
**

**Notes:** A, The Decision Curve Analysis of PFS; B, The Decision Curve Analysis of OS.

**Figure S11.** Kaplan-Meier curve of low and high score of nomograms in patients with colorectal cancer.

**
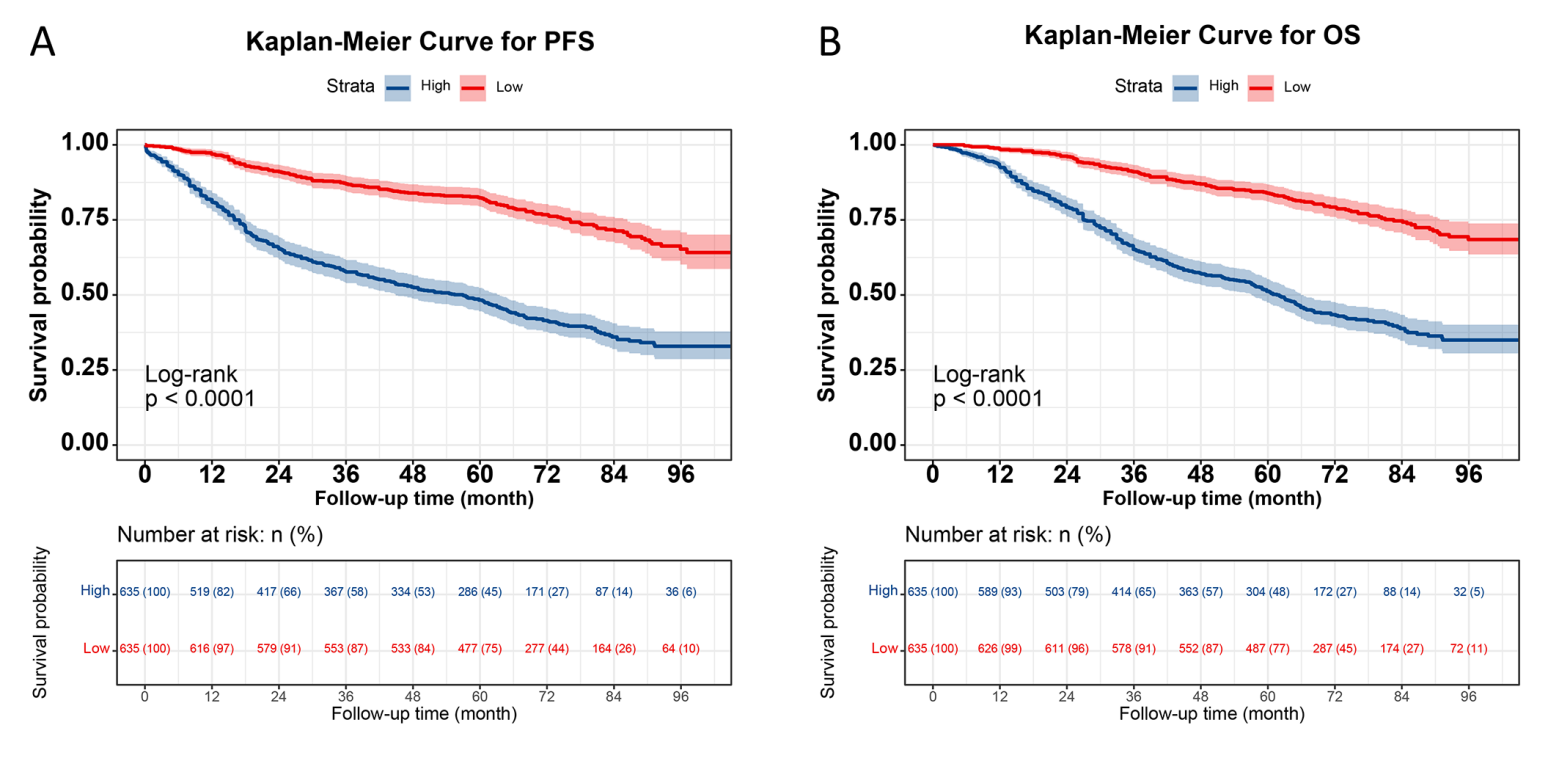
**

**Notes:** A, PFS nomogram; B, OS nomogram. We divided the cohort into high and low-risk groups based on the median risk score obtained from the nomogram. Subsequently, we compared the survival differences between these two groups.

**Table S1** Clinicopathological characteristics of patients with colorectal cancer.

| Clinicopathological characteristics | Overall | Low ApoA-I | High ApoA-I | p |
| --- | --- | --- | --- | --- |
|  | n=1270 | n=275 | n=995 |  |
| Sex (Man) | 804 (63.3) | 222 (80.7) | 582 (58.5) | <0.001 |
| Age (mean (SD)) | 59.22 (12.65) | 59.15 (13.53) | 59.23 (12.41) | 0.921 |
| BMI (median [IQR]) | 22.07 (20.00, 24.44) | 22.39 (20.27, 24.79) | 22.00 (19.95, 24.22) | 0.105 |
| Hypertension (Yes) | 222 (17.5) | 61 (22.2) | 161 (16.2) | 0.026 |
| Diabetes (Yes) | 83 ( 6.5) | 29 (10.5) | 54 ( 5.4) | 0.004 |
| T stage (T3-4) | 952 (75.0) | 230 (83.6) | 722 (72.6) | <0.001 |
| N stage |  |  |  | 0.823 |
| N0 | 715 (56.3) | 151 (54.9) | 564 (56.7) |  |
| N1 | 342 (26.9) | 78 (28.4) | 264 (26.5) |  |
| N2 | 213 (16.8) | 46 (16.7) | 167 (16.8) |  |
| M stage | 120 ( 9.4) | 43 (15.6) | 77 ( 7.7) | <0.001 |
| TNM stage (III-IV) | 591 (46.5) | 138 (50.2) | 453 (45.5) | 0.193 |
| Perineural invasion (Yes) | 130 (10.2) | 28 (10.2) | 102 (10.3) | 0.999 |
| Vascular invasion (Yes) | 220 (17.3) | 55 (20.0) | 165 (16.6) | 0.217 |
| Differentiation (Poor) | 164 (12.9) | 40 (14.5) | 124 (12.5) | 0.418 |
| Location (Rectal cancer) | 647 (50.9) | 98 (35.6) | 549 (55.2) | <0.001 |
| Tumor size (median [IQR]) | 4.78 (2.09) | 5.74 (2.54) | 4.52 (1.86) | <0.001 |
| CEA (median [IQR]) | 3.94 (2.11, 11.28) | 4.90 (2.22, 16.92) | 3.72 (2.09, 9.95) | 0.003 |
| Radiotherapy (%) | 110 ( 8.7) | 13 ( 4.7) | 97 ( 9.7) | 0.012 |
| Chemotherapy (%) | 564 (44.4) | 117 (42.5) | 447 (44.9) | 0.526 |
| Death (Yes) | 517 (40.7) | 148 (53.8) | 369 (37.1) | <0.001 |
| Recurrence (Yes) | 349 (27.5) | 93 (33.8) | 256 (25.7) | 0.01 |
| Length of stay (median [IQR]) | 17.00 (11.00, 21.00) | 18.00 (13.00, 23.00) | 16.00(11.00, 20.00) | <0.001 |
| Hospitalization cost (median [IQR]) | 49886.36 (44834.59, 56372.89) | 52486.85(47234.37, 60828.24) | 48915.19 (44195.04, 55566.12) | <0.001 |

Table Note: CRC, colorectal cancer; BMI, body mass index.

**Table S2** Univariate and multivariate Cox regression analysis of clinicopathological characteristics associated with progression-free survival.

| Characteristic | Progression-free survival | | | |
| --- | --- | --- | --- | --- |
|  | Univariate analysis | | Multivariate analysis | |
|  | HR (95%CI) | P value | HR (95%CI) | P value |
| Age | 1.251 (1.057-1.482) | 0.009 | 1.321 (1.111 - 1.57) | 0.002 |
| BMI |  |  |  |  |
| Normal BMI | 1.208 (0.939-1.555) | 0.141 | 1.186 (0.918 - 1.531) | 0.192 |
| High BMI | 1.328 (1.002-1.76) | 0.048 | 1.399 (1.053 - 1.86) | 0.021 |
| T stage (T3-4) | 2.419 (1.906-3.071) | <0.001 | 1.479 (1.145 - 1.909) | 0.003 |
| N stage |  | <0.001 |  | <0.001 |
| N0 | Ref. |  | Ref. |  |
| N1 | 1.867 (1.528-2.282) | <0.001 | 1.475 (1.197 - 1.818) | <0.001 |
| N2 | 4.122 (3.358-5.06) | <0.001 | 2.792 (2.216 - 3.518) | <0.001 |
| M stage | 5.264 (4.251-6.518) | <0.001 | 3.043 (2.417 - 3.83) | <0.001 |
| Perineural invasion (Yes) | 1.815 (1.431-2.301) | <0.001 | 1.142 (0.874 - 1.493) | 0.33 |
| Vascular invasion (Yes) | 2.064 (1.702-2.503) | <0.001 | 1.203 (0.958 - 1.51) | 0.112 |
| Differentiation (high/medium) | 0.672 (0.534-0.846) | 0.001 | 0.789 (0.621 - 1.002) | 0.052 |
| Tumor size (≥5cm) | 1.199 (1.014-1.417) | 0.033 | 0.954 (0.802 - 1.136) | 0.599 |
| CEA (≥5ng/ml) | 1.916 (1.62-2.266) | <0.001 | 1.467 (1.227 - 1.753) | <0.001 |
| ApoA-I (High) | 0.629 (0.522-0.757) | <0.001 | 0.778 (0.641 - 0.944) | 0.011 |

Table Note: BMI, body mass index.

**Table S3** Univariate and multivariate Cox regression analysis of clinicopathological characteristics associated with overall survival.

| Characteristic | Overall survival | | | |
| --- | --- | --- | --- | --- |
|  | Univariate analysis | | Multivariate analysis | |
|  | HR (95%CI) | P value | HR (95%CI) | P value |
| Age | 1.308 (1.098-1.558) | 0.003 | 1.342 (1.122 - 1.604) | 0.001 |
| T stage | 2.549 (1.981-3.28) | <0.001 | 1.505 (1.15 - 1.971) | 0.003 |
| N stage |  | <0.001 |  | <0.001 |
| N0 | Ref. |  |  |  |
| N1 | 1.86 (1.511-2.29) | <0.001 | 1.488 (1.199 - 1.848) | <0.001 |
| N2 | 4.147 (3.36-5.118) | <0.001 | 2.657 (2.095 - 3.369) | <0.001 |
| M stage | 5.424 (4.368-6.737) | <0.001 | 3.123 (2.475 - 3.94) | <0.001 |
| Perineural invasion (Yes) | 1.769 (1.385-2.261) | <0.001 | 1.1 (0.835 - 1.448) | 0.498 |
| Vascular invasion (Yes) | 2.072 (1.7-2.525) | <0.001 | 1.235 (0.98 - 1.556) | 0.074 |
| Differentiation (high/medium) | 2.072 (1.7-2.525) | <0.001 | 1.235 (0.98 - 1.556) | 0.074 |
| Tumor size (≥5cm) | 1.316 (1.107-1.563) | 0.002 | 1.06 (0.887 - 1.267) | 0.523 |
| CEA (≥5ng/ml) | 1.956 (1.645-2.326) | <0.001 | 1.454 (1.21 - 1.746) | <0.001 |
| ApoA-I (High) | 0.622 (0.514-0.752) | <0.001 | 0.776 (0.637 - 0.945) | 0.012 |

Table Note: BMI, body mass index.
